# Thrombophilia and outcomes of venous thromboembolism in older patients

**DOI:** 10.1016/j.rpth.2022.100015

**Published:** 2022-12-16

**Authors:** Marie Méan, Neal Breakey, Odile Stalder, Lorenzo Alberio, Andreas Limacher, Anne Angelillo-Scherrer, Pierre Fontana, Hans Jürg Beer, Nicolas Rodondi, Drahomir Aujesky, Bernhard Lämmle, Robert Escher

**Affiliations:** 1 Division of Internal Medicine, Lausanne University Hospital, Lausanne, Switzerland; 2 Department of Internal Medicine, Spital Emmental, Burgdorf, Switzerland; 3 CTU Bern, University of Bern, Bern, Switzerland; 4 Service and Central Laboratory of Hematology, Lausanne University Hospital and University of Lausanne, Lausanne, Switzerland; 5 Department of Hematology and Central Hematology Laboratory, Inselspital, Bern University Hospital, University of Bern, Bern, Switzerland; 6 Departement of BioMedical Research, University of Bern, Bern, Switzerland; 7 Division of Angiology and Haemostasis, University Hospitals of Geneva, Geneva, Switzerland; 8 Department of Internal Medicine, Cantonal Hospital of Baden, Baden, Switzerland; 9 Department of General Internal Medicine, Inselspital, Bern University Hospital, University of Bern, Bern, Switzerland; 10 Institute of Primary Health Care, University of Bern, Bern, Switzerland; 11 Center for Thrombosis and Hemostasis, University Medical Center, Mainz, Germany; 12 Haemostasis Research Unit, University College London, UK

**Keywords:** blood coagulation disorders, elderly, recurrence, thrombophilia, venous thromboembolism

## Abstract

**Background:**

Limited data exist on thrombophilic risk factors and clinical outcomes in the elderly with venous thromboembolism (VTE).

**Objectives:**

To describe the prevalence of laboratory thrombophilic risk factors and their association with VTE recurrence or death in a cohort of elderly people with VTE.

**Methods:**

In 240 patients aged ≥65 years with acute VTE without active cancer or an indication for extended anticoagulation, we performed laboratory thrombophilia testing 1 year after the index VTE. Recurrence or death was assessed during the 2-year follow-up.

**Results:**

A total of 78% of patients had ≥1 laboratory thrombophilic risk factor(s). Elevated levels of von Willebrand factor, homocysteine, coagulant activity of factor VIII (FVIII:C), fibrinogen, FIX:C, and low antithrombin activity were the most prevalent risk factors (43%, 30%, 15%, 14%, 13%, and 11%, respectively). Additionally, 16.2% of patients experienced VTE recurrence and 5.8% of patients died. Patients with a von Willebrand factor of >182%, FVIII:C level >200%, homocysteine level >15μmol/L, or lupus anticoagulant had a significantly higher rate of recurrence than those without these risk factors (15.0 vs. 6.1 [*P* = .006], 23.5 vs. 8.2 [*P* = .01], 17.0 vs. 6.8 [*P* = .006], and 89.5 vs. 9.2 [*P* = .02] events per 100 patient-years, respectively). Furthermore, patients with a high fibrinogen level or hyperhomocysteinemia with a homocysteine level ≥30 μmol/L had significantly higher mortality than patients with normal levels (18.5 vs. 2.8 [*P* = .049] and 13.6 vs. 2 [*P* = .002] deaths per 100 patient-years, respectively). After adjustments for relevant confounders, these associations remained unchanged.

**Conclusion:**

Laboratory thrombophilic risk factors are common in elderly people with VTE and allow for the identification of a population at the risk of worse clinical outcomes.

## Essentials

• Blood tests often show a risk of clotting in older patients who have already had a blood clot.
• We performed these tests in 240 patients aged ≥65 years, 1 year after a blood clot.
• Specific blood tests showed a risk of a further blood clot after stopping a blood-thinning therapy.
• The risk of a recurrent blood clot increased with the number of laboratory risk factors present.

## Graphical abstract

**Figure:**
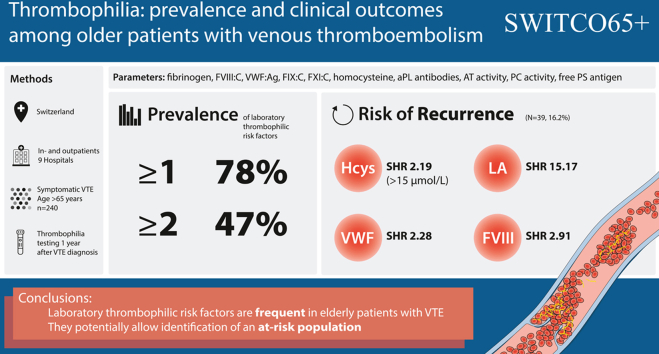

## 1 Introduction

Risk factors for venous thromboembolism (VTE) include clinical conditions and laboratory-based blood parameters. [1] Age is a strong driver of index VTE risk, likely owing, at least in part, to comorbidities, medications, immobilization, changes in the vessel wall, and stasis as part of the Virchow triad. [1] Whether laboratory-based blood parameters influence VTE recurrence and outcomes in elderly patients is unknown. Elderly patients are often excluded from studies owing to comorbidities, short life expectancy, and logistical difficulties concerning data collection. [2]

In the case-control Age and Thrombosis, Acquired and Genetic risk Factors in the Elderly Study (AT-AGE) study, a subgroup of 312 outpatients aged ≥70 years with a first episode of VTE showed an association between high levels of coagulation factors VIII (FVIII) and XI (FXI) and the occurrence of VTE, and another subgroup of 163 patients showed an association between a high level of factor IX (FIX) with first VTE; the associations were calculated by comparing the highest and lowest quartiles (odds ratios of 4.5, 1.7, and 2.4 for FVIII, FXI, and FIX, respectively). [2] People with active malignancy, a history of VTE, or severe cognitive impairment were excluded from that study. No outcome data were reported. In another subgroup of 394 patients from the AT-AGE study, genetic analyses showed a prevalence of 8.6% for factor (F)V Leiden and 2.3% for the prothrombin G20210A polymorphism, and the presence of the FV Leiden variant was associated with a 2.4-fold increased risk of a first unprovoked VTE, but not the prothrombin G20210A polymorphism. [3]

Analysis of 65 patients aged ≥65 years in the Cardiovascular Health Study showed a statistically significant association between levels of FVIII >124% and incident cases of first VTE, whereas no such association was found for FVII or von Willebrand factor (VWF). [4] In a separate analysis of this study population using a nested case-control study design, an FXI level >157% was independently associated with the increased risk of a future first VTE, whereas elevated levels of FIX, factors X (FX), XII (FXII), and XIII (FXIII) were not. [5] Further analyses of this study population showed an association between the FV Leiden variant or the prothrombin G20210A polymorphism and VTE, but not for levels of FII, FV, or homocysteine or for the methylene tetrahydrofolate reductase C677T gene polymorphism. [[6], [7], [8]]

In 2 retrospective studies, the risk of a first VTE increased linearly with increasing levels of FVIII in patients aged >70 years, reaching a 2.4-fold increased risk in people with FVIII >225%, [9] and VTE occurrence was associated with FV Leiden in patients aged >60 years. [10]

In addition, we have previously shown in 354 elderly in- and outpatients with a first unprovoked VTE and a median follow-up period of 30 months that there was no association between FV Leiden and the prothrombin G20210A polymorphism with recurrence [11] and that prohemostatic GAS6 plasma concentrations were independently associated with VTE recurrence and death. [12]

Despite limited data on laboratory risk factors for first VTE in the elderly, it stands to reason that laboratory thrombophilic risk factors may also play a role in the elderly regarding outcomes such as VTE recurrence and death. Therefore, we explored the prevalence of elevated levels of procoagulant factors, such as fibrinogen, FVIII, VWF, FIX, FXI, and homocysteine; the presence of anti–phospholipid (aPL) antibodies including anticardiolipin (aCL), anti–β2-glycoprotein I IgG and IgM antibodies, and lupus anticoagulant (LA); FV Leiden and prothrombin G20210A polymorphism; and low levels of inhibitors of coagulation antithrombin (AT), protein C (PC), and protein S (PS) and the association of these factors with VTE recurrence and death in a prospective cohort of ≥65-year-old people with VTE.

## 2 Methods

### 2.1 Study design, setting, and participants

This study was conducted between September 2, 2009, and December 6, 2013, as part of the Swiss Thromboembolism Cohort (SWITCO65+) study, a prospective multicenter cohort study that assessed long-term clinical outcomes in elderly people with acute VTE from 5 university and 4 high-volume nonuniversity public academic teaching hospitals in Switzerland. The study rationale and full design have been published previously. [[11], [12], [13], [14]] The trial has been registered at http://clinicaltrials.gov (NCT00973596). Briefly, consecutive patients aged ≥65 years with an acute, objectively confirmed symptomatic VTE were prospectively identified in the in- and outpatient services of all participating study sites. Symptomatic pulmonary embolism (PE) was defined as a positive spiral computed tomography or pulmonary angiography, a high probability ventilation-perfusion scan, or proximal deep venous thrombosis (DVT) documented using compression ultrasonography or contrast venography in people with acute chest pain, new or worsening dyspnea, hemoptysis, or syncope. Symptomatic DVT was defined as an acute onset of leg pain or swelling plus incomplete compressibility of a venous segment on ultrasonography or an intraluminal filling defect on contrast venography. Vitamin-K antagonist therapy was the long-term anticoagulant of choice for 95% of patients. The study was approved by the Institutional Review Board at each participating center.

In the present study, laboratory blood parameters, including fibrinogen, factor VIII coagulant activity (FVIII:C), VWF antigen (VWF:Ag), FIX:C, FXI:C, fasting homocysteine, aPL antibodies, AT activity, PC activity, and free PS antigen, were analyzed in 240 people with VTE. Similar to prior studies, [2,3,[14], [15], [16]] blood sampling for these tests was performed after the completion of therapeutic anticoagulation at a follow-up visit 1 year after the index VTE event, and persons with ongoing anticoagulation or active malignancy were excluded. The flow chart of the study population is summarized in Supplementary Figure 1. The total follow-up time was 2 years after thrombophilia testing (median, 718 days; IQR, 521-896 days). To avoid a bias in the choice or duration of therapy during the follow-up, thrombophilia tests were performed independently of the clinical management, and the results were not communicated to the patients or their managing physicians.

### 2.2 Laboratory measurements

Details regarding blood sampling/processing and the methods of measuring thrombophilic factors were described elsewhere. [14] Citrated platelet–poor plasma was used for the following assays: i) FVIII:C, FIX:C, and FXI:C using one-stage coagulation assays using FVIII-, FIX-, and FXI-deficient substrate plasmas, respectively, on a BCS-XP coagulometer according to the manufacturer’s (Siemens) instruction; ii) fibrinogen concentration according to a modified Clauss method using a BCS-XP coagulometer and Multifibren U reagent (Siemens); iii) AT activity as heparin cofactor using Coamatic LR Antithrombin (Chromogenix) on a BCS-XP coagulometer; iv) PC anticoagulant activity using STA-Staclot protein C reagent (Stago) and a BCS-XP coagulometer; v) VWF:Ag using immunoturbidimetry on a BCS-XP device according to the manufacturer’s (Siemens) instruction; vi) free PS antigen using Innovance Free PS Ag reagent on a CS5100 coagulometer (Siemens); vii) aCL and anti–β2-glycoprotein I (aβ2GPI) IgG/ IgM antibodies using HemosIL-AcuStar (Instrumentation Laboratory), a fully automated assay using chemiluminescent technology; and viii) LA screening using Diluted Russel's Viper Venom Test and Partial Thromboplastin Time-Lupus Anticoagulant tests on a BCS-XP coagulometer (Siemens). In case of positive screening test, a confirmation test was done using Staclot-LA (Stago) on a BCS-XP coagulometer. Methods for determining FV Leiden and the prothrombin G20210A polymorphism have been described earlier. [11]

Levels of FVIII:C, VWF:Ag, FIX:C, FXI:C, AT activity, PC activity, and free PS antigen were expressed as a percentage of international standard normal plasma using working standard plasmas calibrated by the manufacturers. The international standard normal plasma contained 100% of the respective factor. The following cut-off values were used to define abnormal levels: FVIII:C >200%, VWF:Ag >182%, FIX:C >134% in women and >138% in men, FXI:C >139% in women and >138% in men, fibrinogen >4.1 g/L, and homocysteine >15 μmol/L (>15-30 μmol/L, moderate; >30-100 μmol/L, intermediate hyperhomocysteinemia). [17] For a better classification, prevalence data for elevated levels of homocysteine >15 μmol/L and >30 μmol/L are given separately as well as for levels of FVIII:C >164% (upper limit of the reference range established in the Central Hematology Laboratory, University Hospital, Bern) and >200%. We chose the latter cut-off because it showed the highest area under the curve in our sensitivity and specificity analyses for VTE recurrence and mortality using FVIII:C cut-offs of 110%, 136%, 164%, and 200%. Similar sensitivity and specificity analyses for homocysteine (using thresholds of 13, 15, 18, 20, and 30 μmol/L) and VWF:Ag (using thresholds of 182% and 187%) led to cut-off values of 15 μmol/L and 182%, respectively. Low AT activity was defined as AT activity <80% in women and <83% in men, PC deficiency as activity <70%, and free PS antigen deficiency as <55% in women and <60% in men; these latter cut-offs were the lower limits of the reference range established in the central laboratory. For aPL antibodies, we used the revised cut-off values as published earlier. [18] We defined positive aPL as aCL IgG level >13.6 U/mL, aCL IgM level >18.7 U/mL, aβ2GPI IgG level >17.4 U/mL, aβ2GPI IgM level >12.0 U/mL, or a positive LA.

### 2.3 Clinical outcomes

The primary outcome was the recurrence of an objectively confirmed, symptomatic VTE, defined as fatal or new nonfatal PE or new proximal or distal DVT. The secondary outcome was all-cause mortality. Primary and secondary outcomes were recorded during the 2-year follow-up period starting at the time of blood sampling 1 year after the index VTE. Follow-up included semiannual contacts alternating between telephone calls and face-to-face evaluations (clinic visits or home visits in house-bound patients) as well as periodic reviews of the patients’ hospital chart.

During each visit/contact, study nurses interviewed patients to obtain information on the date and type of clinical events (recurrent VTE). Reported clinical events and death were crosschecked by reviewing medical charts and interviewing patients’ primary care physicians and family members. Moreover, study nurses obtained information on the cause of death from hospital discharge letters and autopsy reports if available.

A committee of 3 independent, blinded clinical experts classified the cause of death as definitely due to PE, possibly due to PE, due to major bleeding, or due to another cause. [13] Death was judged to be caused by a definite, fatal PE if it was confirmed by autopsy or if it followed a clinically severe PE. Death of a patient who died suddenly without any obvious cause was classified as a possible fatal PE. Final classifications were made on the basis of full consensus of this committee.

### 2.4 Statistical analyses

Baseline characteristics are presented as median and IQR or number and percentage as appropriate. We compared baseline characteristics of patients with and without the presence of any thrombophilia. We defined unprovoked VTE as the absence of major surgery within the previous 3 months, estrogen therapy, and immobilization (fracture or cast of the lower extremity, bed rest >72 hours, or voyage in sitting position for >6 hours) during the last 3 months.

We calculated incidence rates of recurrent VTE and overall mortality for patients with or without a given thrombophilic risk factor using mid two-sided *P* values to test for incidence difference. We compared cumulative incidences using Kaplan-Meier curves and the log-rank test starting from the observation period at the time of blood sampling. We examined associations between laboratory thrombophilic risk factors and time-to-VTE recurrence using competing risk regression accounting for non-VTE–related death as a competing event and the association with death using Cox proportional hazards models. We examined the strength of these associations in further models adjusting for prior VTE and unprovoked VTE in the VTE recurrence model, and additionally for C-reactive protein when calculating the subhazard ratios (SHR) for FVIII and VWF, and for age in the mortality model. Additional adjustment for inflammation using C-reactive protein was performed for the association between elevated fibrinogen levels and overall mortality. Adjustments for FVIII, VWF, and fibrinogen were made using C-reactive protein as a surrogate marker for inflammation as they act as positive acute phase proteins. We examined the interactions of VWF and FVIII in a joint model, with additional adjustment for unprovoked index VTE and prior VTE. We assumed missing and noninterpretable laboratory values to be normal. All analyses were performed using Stata 17 (Stata Corporation).

## 3 Results

As indicated in Supplementary Figure 1, of the original SWITCO65+ cohort, we were able to analyze laboratory data 1 year after the index VTE and clinical follow-up for 2 years in 240 patients. Overall, patients with a laboratory thrombophilic risk factor as compared with those without it were significantly older (median age, 74 vs. 71 years) and more frequently had a low physical activity level (30% vs. 13%, respectively; Table 1). Two hundred thirty-nine patients reported Caucasian race and ethnicity and 1 patient reported African race and ethnicity. At initial recruitment in the SWITCO65+ cohort, majority of patients (67%) had unprovoked VTE and 13% were known for a prior VTE. The median duration of anticoagulant treatment after VTE did not differ between patients with a laboratory thrombophilic risk factor and those without it (187 days [IQR, 126-217 days] vs. 178 days [IQR, 99-204 days]).

**Table 1:** Patient characteristics at the time of index VTE and duration of initial anticoagulation

	All	Any thrombophilia present^a^	No thrombophilia	Missing
	n (%), median (lower; upper quartile)	n (%), median (lower; upper quartile)	n (%), median (lower; upper quartile)	n (%)
**Patient characteristics**	240 (100)	188 (78)	52 (22)	
Patient age (y)	74.0 (69.0;78.8)	74.0 (69.0;79.3)	71.0 (68.0;76.0)	0 (0)
Age ≥80 y	53 (22)	46 (24)	7 (13)	0 (0)
Male	128 (53)	100 (53)	28 (54)	0 (0)
Body mass index >30 kg/m^2^	60 (25)	51 (27)	9 (17)	0 (0)
Arterial hypertension	143 (60)	118 (63)	25 (48)	0 (0)
Low physical activity	63 (26)	56 (30)	7 (13)	0 (0)
Polypharmacy	97 (40)	82 (44)	15 (29)	0 (0)
Diabetes mellitus	35 (15)	31 (16)	4 (8)	0 (0)
**VTE type**				
Unprovoked	161 (67)	130 (69)	31 (60)	0 (0)
No prior VTE	208 (87)	161 (86)	47 (90)	0 (0)
**VTE treatment**				
Anticoagulant treatment duration (d)	186.5 (117.5; 215.3)	187.0 (126.3;217.0)	178.0 (98.5;204.0)	2 (1)

VTE, venous thromboembolism.

a Defined as the presence of at least one thrombophilic factor: antithrombin, protein C, or protein S deficiency; elevated levels of von Willebrand factor antigen (>182%), coagulant activity of factor VIII (FVIII:C) (>200%), FIX:C, FXI:C, homocysteine, and fibrinogen; and the presence of anti–phospholipid antibodies, including lupus anticoagulant, factor V Leiden variant, or prothrombin G20210A polymorphism.

Seventy-eight percent of patients had at least 1 and 47% had ≥2 laboratory-based thrombophilic risk factors (Table 2). Elevated VWF:Ag (thereafter termed VWF) and increased homocysteine were the most prevalent thrombophilic risk factors (43% and 30%, respectively), followed by high FVIII:C (thereafter termed FVIII; 15%), high fibrinogen (14%), high FIX:C (thereafter termed FIX; 13%) and low AT activity (11%). The prevalence of any thrombophilia was not different in patients with unprovoked (130/161 = 81%) vs. provoked VTE (58/79 = 73%; *P* = .24). Similarly, it was not different in those with a first VTE (161/208 = 77%) vs. those with prior VTE before the index event (27/32 = 84%; *P* = .37; Table 1). Anti–phospholipid antibodies including LA were found frequently (total 18%), which were mostly aCL antibodies such as IgG (5%) and IgM (7%) (Table 2). Of the 2 patients with LA, one had only LA and the other additionally had high aCL IgG and IgM levels.

**Table 2:** Prevalence of laboratory-based thrombophilic risk factors in 240 patients with no active cancer and no ongoing anticoagulation 1 year after index VTE.

Thrombophilic risk factor	Prevalence (n/%)	Missing (n/%)^a^
High factor VIII:C (>200%)	36 (15%)	1 (0%)
High factor VIII:C (>164%)	62 (26%)	1 (0%)
Von Willebrand factor antigen (>182%)	103 (43%)	0 (0%)
High factor IX:C (female, >134%; male, >138%)	30 (13%)	1 (0%)
High factor XI:C (female, >139%; male, >138%)	16 (7%)	1 (0%)
High fibrinogen (>4.1g/L)	34 (14%)	1 (0%)
Hyperhomocysteinemia (>15 μmol/L)	71 (30%)	27 (11%)
Intermediate hyperhomocysteinemia (>30 μmol/L)	6 (3%)	27 (11%)
Low **a**ntithrombin activity (female, <80%; male, <83%)^b^	26 (11%)	1 (0%)
Protein C deficiency (<70%)	7 (3%)	6 (3%)
Free **p**rotein S deficiency (female, <55%; male, <60%)	6 (3%)	4 (2%)
Anticardiolipin IgG**–**positive (>13.6 U/mL)	13 (5%)	0 (0%)
Anticardiolipin IgM**–**positive (>18.7 U/mL)	16 (7%)	0 (0%)
Anti**–**β2-**g**lycoprotein I IgG**–**positive (>17.4 U/mL)	7 (3%)	0 (0%)
Anti**–**β2-**g**lycoprotein I IgM**–**positive (>12.0 U/mL)	5 (2%)	0 (0%)
Any antiphospholipid antibody	43 (18%)	0 (0%)
Lupus anticoagulant–positive	2 (1%)	3 (1%)
Factor V Leiden	23 (10%)	11 (5%)
Prothrombin G20210A	9 (4%)	11 (5%)
≥1 thrombophilic risk factors^c^	188 (78%)	-
≥2 thrombophilic risk factors^c^	113 (47%)	-

VTE, venous thromboembolism.

a Missing values out of the total population of 240 patients

b Antithrombin activity (%) and median (IQR, 25%-75%; total range, 78.5%, 74%-81%, and 62%-82%).

c Cut-off levels of 200% and 15 μmol/L were used in the analyses for factor VIII:C and homocysteine, respectively.

**Figure 1:**
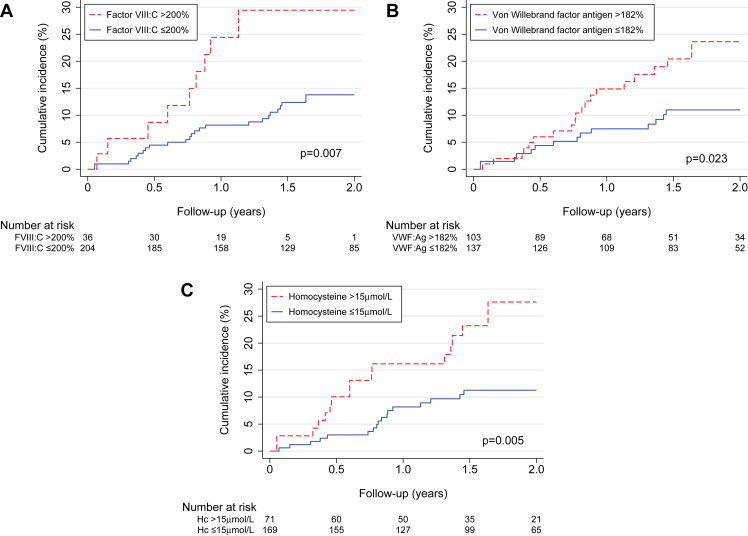
Kaplan-Meier curves with log-rank *P* values for VTE recurrence in patients with elevated factor VIII:C (FVIII:C) level >200% (A), elevated von Willebrand factor >182% (B), and elevated homocysteine level >15 μmol/L (C). Hc, homocysteine; VTE, venous thromboembolism; VWF:Ag, von Willebrand factor antigen

Thirty-nine patients (16.2%) experienced a VTE recurrence during the 2-year follow-up period (Table 3). Patients with a high level of FVIII, a high level of VWF, or elevated homocysteine or LA had a statistically higher incidence rate of recurrent VTE than patients without these risk factors (23.5 vs. 8.2 [*P* = .01], 15.0 vs. 6.1 [*P* = .006], 17.0 vs. 6.8 [*P* = .006], and 89.5 vs. 9.2 [*P* = .02] events per 100 patient-years, respectively; Table 3). When we included age as a covariate in the model, the results did not change markedly. VTE recurrence was significantly more frequent when patients carried ≥2 thrombophilic risk factors (Table 4). The Kaplan-Meier estimates demonstrated that patients with a high level of FVIII had a 2-fold higher 2-year cumulative incidence of recurrent VTE than patients without this risk factor (Figure 1A). The risk increased over time. Similar findings were noted with the presence of elevated VWF or high homocysteine (>15 μmol/L; Figure 1B, C). After adjustment for prior VTE and unprovoked index VTE, elevated FVIII, elevated VWF, and high homocysteine level >15 μmol/L all remained statistically significantly associated with VTE recurrence (Table 5). The findings remained significant for homocysteine level >15 μmol/L when missing data (11%) were excluded and not considered as normal (SHR [95% CI], 2.03 [1.03, 3.98]; data not shown). Additionally, the results remained unchanged for FVIII and VWF when an additional adjustment was performed using elevated C-reactive protein (>5 mg/L) as a surrogate marker for inflammation (SHR [95% CI], 2.45 [1.02, 5.92]; *P* = .046 and SHR [95% CI], 2.35 [1.20, 4.61]; *P* = .01, respectively). By using high FVIII level >200% and VWF level >182% jointly in the models and adjusting the factors for each other, the SHR for each one remained quite substantial, demonstrating a rather independent effect of each one (Supplementary Table 1). Accordingly, only a moderate Pearson correlation of 0.41 between FVIII and VWF was found. In aPL antibody–positive patients, only positive LA was associated with an increased risk of VTE recurrence, but the prevalence of LA was very low.

**Table 3:** Incidence rates of VTE recurrence in 240 patients followed up for 2 years after thrombophilia testing.^a^

Thrombophilic risk factor	Present		Absent		
	Number of events/patients	Events per 100 patient-y (95% CI)	Number of events/patients	Events per 100 patient-y (95% CI)	*P* value
High factor VIII:C (>200%)	9/36	23.5 (12.2-45.2)	30/204	8.2 (5.7-11.7)	.01
Von Willebrand factor antigen (>182%)	24/103	15.0 (10.0-22.3)	15/137	6.1 (3.7-10.2)	.006
High factor IX:C	4/30	7.5 (2.8-19.9)	35/210	10.0 (7.2-13.9)	.62
High factor XI:C	2/16	8.9 (2.2-35.7)	37/224	9.7 (7.0-13.3)	.99
High fibrinogen	7/34	17.1 (8.1-35.8)	32/206	8.8 (6.2-12.4)	.13
Hyperhomocysteinemia (>15 μmol/L)	19/71	17.0 (10.8-26.6)	20/169	6.8 (4.4-10.6)	.006
Low antithrombin activity	4/26	10.5 (3.9-28.0)	35/214	9.5 (6.8-13.3)	.81
Protein C deficiency	0/7	0	39/233	10.0 (7.3-13.7)	.21
Free protein S deficiency	0/6	0	39/234	9.9 (7.2-13.6)	.33
Anticardiolipin IgG–positive	3/13	15.6 (5.0-48.2)	36/227	9.3 (6.7-12.9)	.40
Anticardiolipin IgM–positive	4/16	15.0 (5.6-40.0)	35/224	9.2 (6.6-12.9)	.36
Anti–β2-glycoprotein I IgG–positive	2/7	15.4 (3.8-61.4)	37/233	9.4 (6.8-13.0)	.49
Anti–β2-glycoprotein I IgM–positive	1/5	12.4 (1.7-88.1)	38/235	9.6 (7.0-13.2)	.73
Lupus anticoagulant–positive	2/2	89.5 (22.4-357.9)	37/238	9.2 (6.7-12.7)	.02
Factor V Leiden	2/23	4.8 (1.2-19.3)	37/217	10.2 (7.4-14.0)	.31
Prothrombin G20210A	0/9	0	39/231	10.1 (7.4-13.8)	.15

VTE, venous thromboembolism.

a Overall, 39 out of 240 patients experienced VTE recurrence (9.6 events per 100 patient-y; 95% CI, 7.0-13.2)

**Table 4:** Comparison of incidence rates of VTE recurrence between patients with 1 or ≥2 thrombophilic risk factors and no thrombophilic risk factor.

Number of thrombophilic risk factors	Number of patients	Number of events/ patient-y	IR (95% CI) per 100 patient-y	*P* value
0 thrombophilic risk factors	52	4/97	4.1 (1.5-11.0)	-
1 thrombophilic risk factor	75	10/130	7.7 (4.1-14.3)	.30
≥2 thrombophilic risk factors	113	25/178	14.1 (9.5-20.8)	.01

IR, incidence rate; VTE, venous thromboembolism.

**Table 5:** Association of thrombophilic risk factors with clinical outcomes after adjustments

	VTE recurrence			All-cause mortality		
	Unadjusted	Adjusted^a^		Unadjusted	Adjusted^a^	
Thrombophilic factor	SHR (95% CI)		*P* value^a^	HR (95% CI)		*P* value^a^
High factor VIII:C (>200%)	2.84 (1.32;6.09)	2.91 (1.28;6.61)	.01	b	b	
Von Willebrand factor antigen (>182%)	2.40 (1.26;4.57)	2.28 (1.20;4.35)	.01	1.93 (0.67;5.55)	1.72 (0.58;5.10)	.33
High factor IX:C	0.79 (0.27;2.28)	0.74 (0.26;2.13)	.58	b	b	
High factor XI:C	0.90 (0.22;3.64)	0.79 (0.20;3.18)	.75	1.32 (0.17;10.11)	1.53 (0.20;11.93)	.68
High fibrinogen	1.70 (0.75;3.83)	1.67 (0.75;3.72)	.21	8.02 (2.66;24.21)	7.93 (2.66;23.67)	<.001
Hyperhomocysteinemia (>15 μmol/L)	2.49(1.34;4.65)	2.19 (1.14;4.21)	.02	0.94 (0.30;3.01)	0.81 (0.25;2.64)	.73
Hyperhomocysteinemia (>30 μmol/L)	b	b		6.46 (1.44;28.91)	5.60 (1.22;25.75)	.03
Low **a**ntithrombin activity	1.12 (0.39;3.21)	1.06 (0.37;3.02)	.92	b	b	
Protein C deficiency	b	b		b	b	
Free **p**rotein S deficiency	b	b		b	b	
Anticardiolipin IgG**–**positive	1.68 (0.55;5.16)	1.95 (0.61;6.20)	.26	b	b	
Anticardiolipin IgM**–**positive	1.65 (0.59;4.63)	1.75 (0.62;4.95)	.29	b	b	
Anti**–**β2-**g**lycoprotein I IgG**–**positive	1.67 (0.39;7.13)	1.90 (0.42;8.58)	.40	b	b	
Anti**–**β2-**g**lycoprotein I IgM**–**positive	1.33 (0.18;10.03)	1.27 (0.17;9.49)	.82	b	b	
Lupus anticoagulant**–**positive	8.96 (5.02;16.00)	15.17 (5.33;43.16)	<.001	13.47 (1.72;105.68)	23.57(2.57;216.37)	.005
Factor V Leiden	0.47 (0.11;1.98)	0.43 (0.10;1.77)	.24	0.66 (0.09;5.03)	0.67 (0.09;5.16)	.71
Prothrombin G20210A	b	b		3.49 (0.78;15.63)	3.39 (0.76;15.21)	.11
≥1 thrombophilic risk factor(s)	2.73 (0.97;7.72)	2.58 (0.92;7.27)	.07	3.94 (0.52;30.14)	3.55 (0.46;27.50)	.23
≥2 thrombophilic risk factors	2.24 (1.16;4.31)	2.11 (1.10;4.05)	.03	1.60 (0.55;4.60)	1.48 (0.51;4.32)	.47

HR, hazard ratio; SHR, subhazard ratio; VTE, venous thromboembolism.

a Adjusted for prior VTE and unprovoked index VTE for VTE recurrence and adjusted for age for mortality; *P* values based on adjusted models. Subhazard ratios were calculated for VTE recurrence in order to account for the competing risk of non–VTE-related death.

b Not estimable because there was no event.

**Figure 2:**
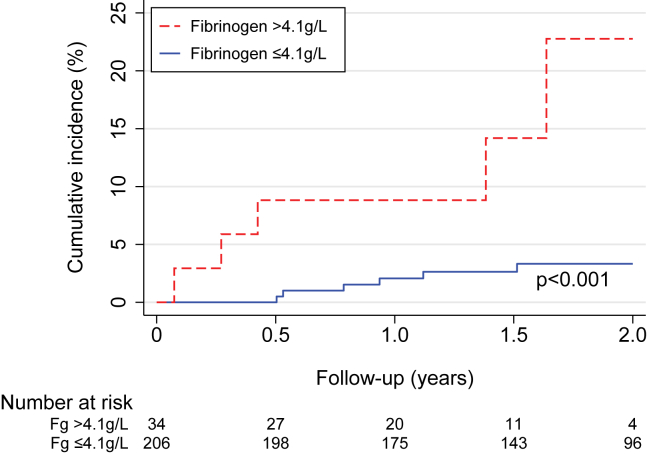
Kaplan-Meier curve with a log-rank *P* value for overall mortality in patients with fibrinogen level >4.1 g/L. Fg, fibrinogen

During the 2-year follow-up period, 14 patients died. The causes of death were as follows: 1 definite PE, 3 possible PE, 4 major bleedings, 1 acute coronary syndrome, 1 sepsis, 2 cancers diagnosed after laboratory thrombophilia work-up, and 2 deaths due to unknown causes. The number of deaths was small. However, it is notable that patients with high fibrinogen levels (>4.1 g/L) had a significantly higher death rate than patients without this risk factor (13.6 vs. 2.0 deaths per 100 patient-years; *P* = .002) (Supplementary Table 2; see Kaplan-Meier estimates in Figure 2) as well as when adjusted for age (Table 5; hazard ratio [HR], 7.93; 95% CI, 2.66-23.67; *P* = <.001) and additionally for inflammation (C-reactive protein level >5 mg/L; HR, 7.02; 95% CI, 2.16-22.79; *P* = .001). Causes of death in these 6 patients with high fibrinogen were possible PE (2 patients), acute coronary syndrome, sepsis, cancer, and unknown (1 patient each). Furthermore, patients with considerably high levels of homocysteine (>30 μmol/L) had a higher incidence rate of death than those with a normal homocysteine level (18.5 vs. 2.8 deaths per 100 patient-years; *P* = .049; Supplementary Table 2), also after adjusting for age (Table 5; HR, 5.60; 95% CI, 1.22-25.75; *P* = .03) or as a sensitivity analysis, by excluding missing data and not considering them as normal (*P* = .03; data not shown). The only death with LA was due to possible PE.

## 4 Discussion

Laboratory thrombophilia was common in our cohort of elderly patients aged ≥65 years with VTE. High levels of FVIII, VWF, fibrinogen, FIX, FXI, and homocysteine and low AT activity were the most prevalent thrombophilic risk factors. In this cohort, some laboratory parameters identified a population at risk of VTE recurrence and death. Our data show that the risk of VTE recurrence more than doubled in the presence of elevated levels of FVIII, VWF, or homocysteine and that the risk of death was more than 6-fold higher in the presence of elevated levels of fibrinogen. Moreover, despite a very low prevalence, LA was also associated with VTE recurrence and death.

### 4.1 Prevalence of laboratory indicators of hypercoagulability

To our knowledge, only few prior studies addressed the prevalence of thrombophilic risk factors among persons aged ≥65 years with VTE, mostly in subgroups only. [2,4,[9], [10], [11],^19^] Most studies focused on isolated or selected thrombophilic risk factors in relation to a first VTE. Only 1 study was prospective and addressed VTE recurrence. [11]

In our cohort of older patients, 78% of patients had ≥1 laboratory thrombophilic risk factor. Limited comparable data exist in the elderly; however, among the 146 patients aged ≥70 years from the MAISTHRO registry, 42.5% of those with a first VTE had any thrombophilic risk factor, [19] and in a cohort of 474 younger people with a first acute VTE, this proportion was 67%. [15] From the available data, there is a high prevalence of thrombophilic risk factors in people with VTE consistent throughout all age categories, including the elderly.

Elevated levels of VWF, FVIII, homocysteine, and fibrinogen were the most prevalent thrombophilic risk factors in our patients. The prevalence of an elevated homocysteine level was very high (30%), substantially higher than the prevalence previously reported in a younger population (18%). [15] The levels of homocysteine, fibrinogen, VWF, and FVIII have been shown to increase with advancing age. [20,21] Regarding homocysteine, it has been hypothesized that dietary-induced deficiencies of folate and cyanocobalamin could contribute to its elevated levels in the elderly. [21] On the other hand, fibrinogen, VWF, and FVIII are acute phase reactants and might be surrogate markers for an underlying inflammatory or neoplastic disorder. However, in younger people with a first VTE, it has been shown that after adjusting for inflammation using C-reactive protein as a surrogate marker, the elevated FVIII levels were independent of any inflammation. [22] We made the same observation in our elderly cohort. Although we excluded people with known active cancer from the analysis, we cannot exclude the possibility of an underlying occult malignancy. However, as only 2 patients died from cancer during the 2-year follow-up, a high prevalence of undiagnosed neoplastic disorders is unlikely.

We found elevated FIX and FXI in 13% and 7% of patients, respectively. We did not find comparable data for the elderly in the literature. Furthermore, a combined prevalence of endogenous anticoagulant (PS, PC, and AT) deficiencies of 16.25% was found in our study, with the highest prevalence for decreased AT (11%). This is surprising, as it is clearly higher than in a smaller subgroup analysis of the MAISTHRO registry in the patients aged ≥70 years, in which the findings for AT, PC, and PS deficiency were 1.4%, 0%, and 1%, respectively, [19] and it is also higher than that in younger patients with a mean age of 45 years (5%). [15] In the general population, the prevalence is <0.5%. [23] A detailed analysis of the low AT activity found in our study population showed a high median of 78.5% with an IQR between 74% and 81% (Table 2). Three patients only had AT levels ≤70%, with the lowest level being 62%. A likely explanation for the antithrombin activities mostly moderately below the normal range in our elderly population is consumption or loss of the anticoagulant factor by renal (nephrotic syndrome) or gastrointestinal (protein-losing enteropathy) disease.

### 4.2 Clinical outcomes

In our analysis, among elderly people with VTE, elevated FVIII clotting activity, elevated VWF, increased homocysteine, and presence of LA were associated with VTE recurrence, and elevated fibrinogen or homocysteine levels >30 μmol/L were associated with all-cause mortality, indicating a potential prognostic influence of these thrombophilic risk factors. The presence of ≥2 laboratory thrombophilic risk factors significantly increased the incidence rate of VTE recurrence.

The association of high levels of FVIII or presence of LA with VTE recurrence and the increasing recurrence risk over time is consistent with previous observations in younger patients. [16,24] A small interventional study of 34 younger patients suggested a benefit for extended anticoagulation in such patients. [25] However, confirmatory data for this intervention in larger cohorts or in the elderly are lacking.

It has been shown that FVIII levels are partly positively and independently influenced by age and the plasma concentration of fibrinogen; importantly, however, in that study, adjustment for fibrinogen has not changed the observed association between elevated FVIII levels with a first VTE in the elderly. [9] Looking for underlying inflammation as a cause of elevated FVIII and VWF and as the cause for the observed association with VTE recurrence, and by using C-reactive protein as a surrogate marker for inflammation and adjusting our findings for it, we found that the results were unchanged. Both FVIII and VWF remained associated with VTE recurrence. Considering FVIII, it is important to look simultaneously at VWF, as VWF acts as a transport protein for FVIII and is critical for initiation of thrombosis. [26] Elevated FVIII has been noted in 33.7% of patients in a subgroup analysis of the MAISTHRO registry, [19] and VWF in the highest quartile was found in the small patient group enrolled in the Cardiovascular Health Study. [4] In that study, FVIII and VWF were independently associated with a first VTE. For both VWF and FVIII, high plasma levels were found in our study (43% and 15%, respectively). Not unexpectedly given their biological relationship, both were associated with VTE recurrence. Importantly, this effect was rather independent for each of the factors. In our study, both factors represent very substantial risk factors for VTE recurrence in the elderly, and one might consider including them in risk assessment for VTE recurrence in this population.

To our knowledge, no study examined the association between high homocysteine and the risk of VTE recurrence specifically in the elderly. [27,28] Our results indicate a significantly increased risk of VTE recurrence in patients with homocysteine levels >15 μmol/L. These findings are in line with a recent analysis of 2 large cohorts of younger women showing an association of homocysteine levels with a future first VTE. [29] Whether homocysteine is merely a surrogate marker of increased VTE recurrence risk and whether the risk can be lowered using folic acid and vitamins B12 and B6 to reduce homocysteine levels remains to be demonstrated in this age group. In younger patients with a median age of 56.4 years, a multicentric study showed no effect of a substitution with folic acid, vitamin B6, and vitamin B12 on VTE recurrence when treating patients with homocysteine levels ≥8.5 μmol/L (≥8.5 μmol/L to ≥12.6 μmol/L according to the participating center). [30]

The mortality rate was low, precluding any firm conclusion. It is of note, however, that previous studies have found an association with death for elevated homocysteine in a younger population (median age of 47.6 years) with a history of VTE (HR, 1.98; 95% CI, 1.07-3.48) [31] for elevated fibrinogen in a large population–based meta-analysis for arterial vascular and nonvascular mortality [32] and for LA in younger people with VTE. [33]

Our study has several potential limitations. Firstly, keeping with similar studies, [2,15,16] laboratory analyses were not performed at the time of the first VTE event or when oral anticoagulation was stopped but were performed after 1 year, with outcome analyses starting at this time point. By doing so, one might hypothesize that the patients at higher risk were excluded, ie, patients that died within the first year and patients with a clinical high-risk situation and ongoing anticoagulation. As in those other studies, [2,15,16] however, there are reasons to do so. As inflammation and acute phase reaction at the time of acute VTE diagnosis increase the levels of acute phase reactants such as fibrinogen, VWF, and FVIII and as anticoagulation with vitamin-K antagonists decreases vitamin-K–dependent factors including FIX, PS, and PC and does not allow measurement for LA, the time point chosen might even be a strength by eliminating biological confounding situations. [23,34] It has to be mentioned that since the time of our study enrolment and follow-up period, direct oral anticoagulants have become the first-line drugs for treating venous thromboembolism, and for rivaroxaban and apixaban, recommendations for extended anticoagulation at reduced doses have been made. [35,36] Secondly, although we excluded people with cancer, our study still enrolled people with provoked index VTE and prior VTE who may have a different risk profile of VTE recurrence. Thirdly, the fact that no recurrence occurred within 12 months following the index VTE after a median anticoagulation duration of about 6 months (Table 1) might suggest that our study population consisted of mainly low-risk people for VTE recurrence. Finally, most of the patients were of Caucasian origin, and the findings cannot be extended to other populations.

In conclusion, laboratory thrombophilic risk factors are frequent in elderly people with VTE. They potentially allow identification of a population at risk of worse clinical outcome. Whether patients with high levels of VWF, FVIII, and homocysteine or with an LA might especially benefit from prolonged secondary prophylactic anticoagulation will need further prospective randomized trials.
