# Supplementary material for: Thrombophilia and outcomes of venous thromboembolism in older patients
Source: Res Pract Thromb Haemost. 2022 Dec 16;7(1):100015. doi: 10.1016/j.rpth.2022.100015 (PMC10031374; doi:10.1016/j.rpth.2022.100015)
Supplement: Supplementary Figure 1 [file mmc1.pdf]

Supplementary Figure 1: Flow chart

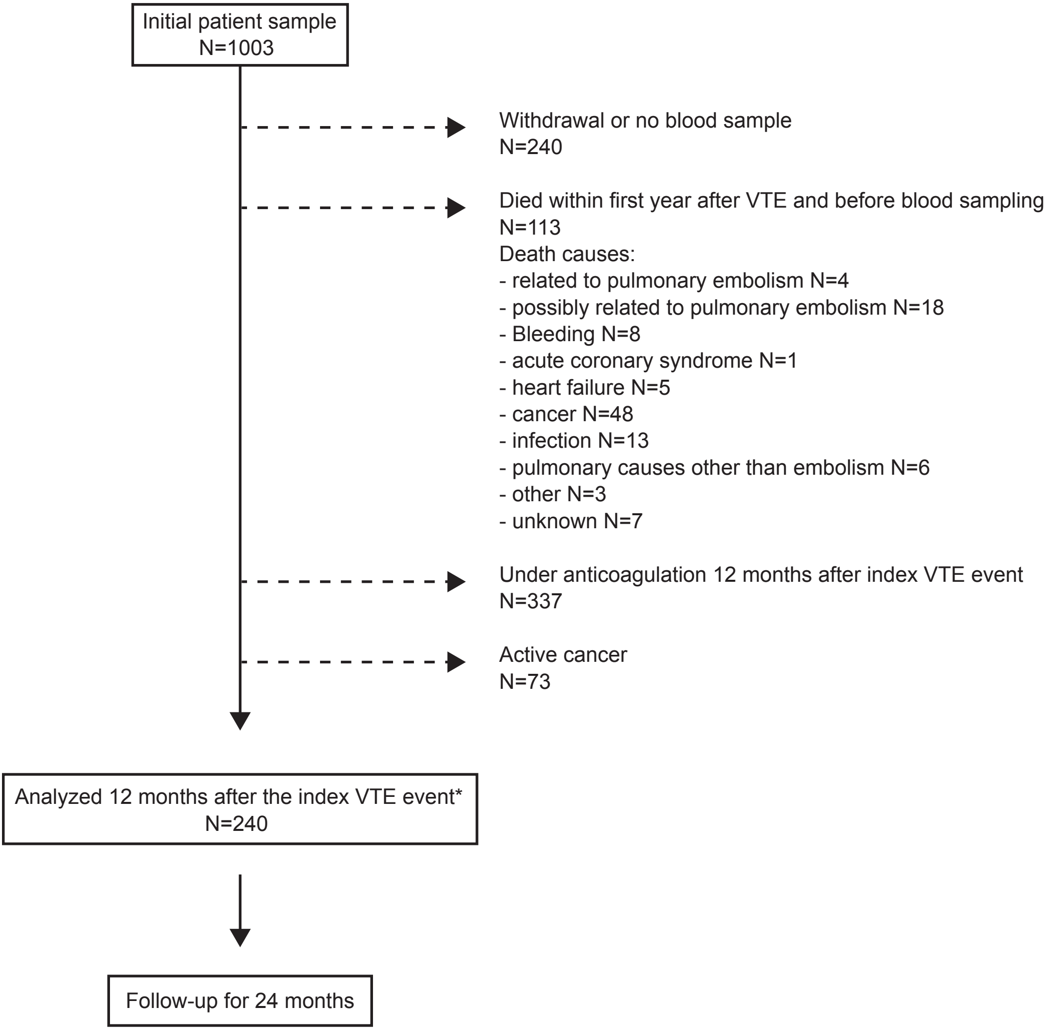

*The median time interval between cessation of anticoagulation and laboratory testing was 176.5 days with an interquartile range of 150-232.5 days.
