# Supplementary material for: Thrombophilia and outcomes of venous thromboembolism in older patients
Source: Res Pract Thromb Haemost. 2022 Dec 16;7(1):100015. doi: 10.1016/j.rpth.2022.100015 (PMC10031374; doi:10.1016/j.rpth.2022.100015)
Supplement: Supplementary Table 1 [file mmc2.docx]

**Supplementary Table 1. VTE recurrence in a joint model using FVIII:C >200% and**

**VWF >182%.**

|  | Joint model |  | Adjusted joint model |  |
| --- | --- | --- | --- | --- |
|  | SHR (95%-CI) | p-value | SHR (95%-CI) | p-value |
|  |  |  |  |  |
| Number of patients | N = 240 |  | N = 240 |  |
| Number of events | N = 39 |  | N = 39 |  |
| **FVIII:C >200%** | **2.02 (0.94;4.36)** | **0.072** | **2.10 (0.91;4.84)** | **0.081** |
| **VWF >182%** | **2.02 (1.04;3.92)** | **0.038** | **1.90 (0.97;3.72)** | **0.060** |

Model includes FVIII:C (>200%) and VWF (>182%) binary variables and is additionally adjusted for unprovoked index VTE and prior VTE.
