# Supplementary material for: Thrombophilia and outcomes of venous thromboembolism in older patients
Source: Res Pract Thromb Haemost. 2022 Dec 16;7(1):100015. doi: 10.1016/j.rpth.2022.100015 (PMC10031374; doi:10.1016/j.rpth.2022.100015)
Supplement: Supplementary Table 2 [file mmc3.docx]

**Supplementary Table 2. Incidence rates of death from any cause in 240 patients within 2 years of thrombophilia testing.***

| Thrombophilic risk factor | Present |  | Absent |  |  |
| --- | --- | --- | --- | --- | --- |
|  | Number of events/patients | Events per 100 patient-years  (95%-CI) | Number of  events/patients | Events per 100 patient-years  (95%-CI) | p-value |
| High factor VIII:C (>200%) | 0/36 | 0 | 14/204 | 3.5 (2.1-6.0) | 0.215 |
| Von Willebrand antigen (>182%) | 8/103 | 4.4 (2.2-8.9) | 6/137 | 2.3 (1.0-5.1) | 0.231 |
| High factor IX:C | 0/30 | 0 | 14/210 | 3.7 (2.2-6.2) | 0.129 |
| High factor XI:C | 1/16 | 3.9 (0.5-27.6) | 13/224 | 3.1 (1.8-5.4) | 0.762 |
| High fibrinogen | 6/34 | 13.6 (6.1-30.3) | 8/206 | 2.0 (1.0-4.0) | 0.002 |
| Hyperhomocysteinemia (>15 µmol/L) | 4/71 | 3.0 (1.1-8.1) | 10/169 | 3.2 (1.7-6.0) | 0.948 |
| Hyperhomocysteinemia (>30 µmol/L) | 2/6 | 18.5 (4.6-74.0) | 12/234 | 2.8 (1.6-4.9) | 0.049 |
| Low Antithrombin activity | 0/26 | 0 | 14/214 | 3.5 (2.1-5.9) | 0.257 |
| Protein C deficiency | 0/7 | 0 | 14/233 | 3.3 (1.9-5.6) | 0.600 |
| Free Protein S deficiency | 0/6 | 0 | 14/234 | 3.3 (1.9-5.5) | 0.692 |
| Anticardiolipin IgG positive | 0/13 | 0 | 14/227 | 3.3 (2.0-5.6) | 0.519 |
| Anticardiolipin IgM positive | 0/16 | 0 | 14/224 | 3.4 (2.0-5.7) | 0.386 |
| Anti-β2-Glycoprotein I IgG positive | 0/7 | 0 | 14/233 | 3.3 (1.9-5.5) | 0.625 |
| Anti-β2-Glycoprotein I IgM positive | 0/5 | 0 | 14/235 | 3.2 (1.9-5.5) | 0.741 |
| Lupus anticoagulant positive | 1/2 | 36.6 (5.2-259.6) | 13/238 | 3.0 (1.7-5.1) | 0.087 |
| Factor V Leiden | 1/23 | 2.2 (0.3-15.8) | 13/217 | 3.3 (1.9-5.6) | 0.800 |
| Prothrombin G20210A | 2/9 | 10.4 (2.6-41.4) | 12/231 | 2.8 (1.6-5.0) | 0.144 |
| ≥1 thrombophilic risk factors | 13/188 | 3.8 (2.2-6.6) | 1/52 | 1.0 (0.1-7.0) | 0.156 |
| ≥2 thrombophilic risk factors | 8/113 | 4.0 (2.0-8.0) | 6/127 | 2.5 (1.1-5.6) | 0.398 |

*Overall, 14/240 patients suffered death from any cause (3.2 events per 100 patient-years ; 95%-CI 1.9-5.4).
